# The Work of Well‐Being: How People Work to Support Their Well‐Being After Stroke

**DOI:** 10.1111/hex.70807

**Published:** 2026-08-04

**Authors:** Claire Ibell‐Roberts, Felicity Bright

**Affiliations:** ^1^ AUT Centre for Person Centred Rehabilitation Research Auckland University of Technology Auckland New Zealand

**Keywords:** activity, patient experience, qualitative, self‐management, stroke, well‐being

## Abstract

**Introduction:**

Stroke can have a range of impacts on a person's life. Previous research has highlighted the need for greater attention to well‐being, with people with stroke reporting that well‐being is not well addressed in stroke services and therefore remains an area of unmet need. While previous research has considered how services address well‐being, this study focused on those with stroke, exploring the work they did to maintain and enhance their well‐being while receiving care from stroke services. It also examined how stroke services appeared to influence this work.

**Materials and Methods:**

Underpinned by Interpretive Description methodology, interviews were conducted with 37 people impacted by stroke (24 people with stroke, 13 family members) across the North Island of Aotearoa New Zealand. Data were analysed using directed content analysis guided by Normalisation Process Theory.

**Results:**

People described undertaking significant work to support their well‐being whilst receiving care from stroke services. We identified six interwoven forms of work which involved ‘inward work’—sense‐making, emotional work and reconnecting work—and ‘outward work’—navigating health services, advocating for needs and doing rehabilitation. All were influenced by a person's network of relationships. People impacted by stroke perceived that the depth of this work was unseen by health professionals and described how stroke services could act to either reduce or exacerbate the work that they were doing for their well‐being.

**Conclusions:**

These findings show how people actively work to sustain their well‐being whilst receiving care from stroke services. By examining how stroke services influence this ‘well‐being work’, we highlight areas where health services may focus efforts to support well‐being. Health professionals can acknowledge the work that people are doing and foster the resources they already hold, such as supporting people to engage in roles, relationships, cultural practices and social contexts that support their well‐being from early in care. By doing so, health professionals and service leaders may set a foundation to better support people's well‐being both in the moment and in the long‐term.

**Patient or Public Contribution:**

People with stroke, family members, and people who provide support to people with stroke, and health professionals set priorities for this research. They advised on study conduct and have provided feedback on wider findings from the research.

## 1 Introduction

Stroke, experienced by approximately 9000 people each year in Aotearoa New Zealand (referred to hereinafter as Aotearoa) [1], can have a range of impacts on a person's life. One area that has been identified as worthy of attention is the area of well‐being [2]. People with stroke report that well‐being is not consistently addressed in stroke services and remains an area of unmet need [3, 4]. Health professionals agree. Many acknowledge the importance of supporting well‐being, but describe feeling constrained in their ability to do so [5]. Well‐being is multifaceted, dynamic and varied across contexts [6]. What constitutes well‐being is unique to each individual, influenced by personal traits, social circumstances and worldview [7]. As such, providing a succinct definition that captures the essence of well‐being is challenging. Instead, it may be better understood through its key characteristics [8]. In previous work, we have argued that while well‐being is unique to each person and their family, key elements consistently arise. It is deeply relational, grounded in connections with others, and incorporating a sense of self and identity, finding balance in the present and holding hope for the future [9]. Well‐being is culturally grounded, with Māori, the indigenous peoples of Aotearoa, highlighting the importance of deep connections to people and places of meaning, and to one's cultural identity. Wairua (spiritual sense), upholding of mana (inherent standing) and rangatiratanga (autonomy and sovereignty) were also described as core elements of well‐being for many Māori [9]. Such elements can be disrupted by stroke, for example through a loss of connection to self‐identity, to meaningful roles, activities, cultural practices, people and places [9, 10]. People are often grappling with challenges to their well‐being whilst receiving care from stroke services and, as such, there is opportunity, and need, for health professionals and stroke services to support well‐being in this time [3, 11, 12].

This study forms part of a larger research project that sought to understand what is important for well‐being after stroke and to improve how it is addressed in stroke services. One notion that consistently came through the interviews with people impacted by stroke was the depth of work that they were doing to support their well‐being, often from very early post‐stroke. Such work appeared largely unseen in the context of stroke services. We draw on Corbin and Strauss’ concept of ‘work’, which encompasses the tasks undertaken by an individual or group, how and where those tasks are performed, and the consequences, challenges and problems associated with carrying them out. While ‘work’ is often associated with physical activities, this sociological concept has been used to explore how people manage the impacts of illness on their sense of self through a form of work called biographical work [13], and to understand how people manage the burden of healthcare following stroke or chronic illness, recognising this treatment burden is a form of work [12]. However, there has been limited exploration of the work that people do to support their well‐being more broadly following stroke. Several authors have highlighted the challenging nature of life early post‐stroke—a time when support from stroke services is typically most intensive. People are commonly coping with shock, making sense of their new reality, adjusting to changed abilities and navigating altered perceptions of their capacity and self‐identity [11, 14, 15, 16]—all activities which indicate forms of work that are undertaken to support well‐being. Our research examines this further, seeking to develop deeper understandings of the work that people do while under the care of stroke services, to make such work more visible and provide opportunities for it to be recognised and supported. As such, the aims of this present study are to identify the work undertaken by people impacted by stroke to maintain and enhance their well‐being whilst receiving care from stroke services, and to examine how stroke services appeared to influence this work.

## 2 Materials and Methods

This qualitative study was underpinned by Interpretive Description [17], a methodology which explores and interprets phenomena while remaining situated within the practice context.

### 2.1 Participants and Recruitment

We sought to engage with people impacted by stroke across Aotearoa. People were eligible to participate if they:

1. Had experienced a stroke, were over 18 years of age and were able to communicate with the researchers in English, with support provided for those with cognitive and communication impairments.
2. Were a family or whānau (wider kinship network) member of a person with stroke and were over 18 years of age (referred to as ‘family’ throughout this paper).

Recruitment had multiple approaches. First, we recruited through two public stroke services covering urban and rural areas in the North Island, which were study sites for the larger study that explored health service delivery as well as patient and staff experience. To gather wider representation, we also advertised the study through national stroke support organisations and leaders in Māori stroke support groups, sharing online notices and inviting participation in the study. We purposively sampled for a diversity of participants in terms of age, impacts of stroke, geographical location and equal representation of Māori and non‐Māori.

Potential participants were provided with an information sheet and verbal information about the study and were given time to consider participation. All were encouraged to invite any support people they wished to include. Informed consent was gained prior to data collection.

### 2.2 Data Collection

Semi‐structured interviews were conducted with a total of 37 people impacted by stroke (people with stroke, family, or carers). Interviews with individuals (*n* = 13), dyads involving the person with stroke and a family member (*n* = 18 people across nine dyads) and small groups (*n* = 6 people across two groups) were conducted in person and online. One small group involved the person with stroke, a family member and a carer; the other involved two people with stroke and a family member of one of these people.

Interview guides were developed (see Appendix A) which explored people's understandings of well‐being, their experiences of well‐being post‐stroke including what influenced their well‐being, their reflections on how it was addressed within their stroke care and how care could be enhanced to support people with stroke. Interviews with participants recruited through the Māori stroke organisation were led by a Māori researcher following tikanga (Māori cultural practices and protocols of engagement). Interviews with all other people with stroke were conducted by a Pākehā (non‐Māori, of European descent) researcher. Individual interviews lasted between 60 and 90 min, while small group interviews lasted between 60 and 120 min. All interviews were audio‐recorded and transcribed.

### 2.3 Ethical Approval

Ethical approval was obtained both from the University Ethics Committee (21/223), and from recruiting localities.

### 2.4 Data Analysis

Data analysis commenced with a process of familiarisation through multiple readings of transcripts and listening to associated recordings. We wrote analytical memos which sought to understand how people perceived their well‐being was impacted while in stroke services. Through this process, it became clear that people were doing significant ‘work’ to support their well‐being. This prompted the need for a sensitising analytic lens to attune to the varying layers of work that people appeared to be doing.

We drew on Normalisation Process Theory (NPT) as a framework to attune to such work. NPT explains how new practices become embedded into a person's everyday life, and the factors that might support or prevent this from happening [18]. It has four components: coherence, cognitive participation, collective action and reflexive monitoring [18]. These components can help attune to the work that people do to normalise new ways of being and doing, which in this context relates to adjusting to the impacts of stroke. While the original writing on NPT references the four constructs above, we drew on how Makela et al. [19] framed these in their study exploring the work of older people and caregivers in managing acute health events. They discuss the constructs of sense‐making work, relational work, enacting work and appraising work. We modified this framework to provide a sensitising device to understand how people worked to support their well‐being after stroke (see Appendix B). Using directed content analysis [20], data were coded deductively into each of the four categories outlined in our framework. Memo writing was used to capture new inductive insights, as it became clear that data did not always directly align with the constructs of our framework. For example, relational aspects of work permeated all categories rather than being easily distinguished from sense‐making, enacting and reflection work. Through regular analytical discussions within the research team, the analysis evolved from the four constructs of our framework into six core areas of work, which are presented below.

### 2.5 Author Positionality

As qualitative research informed by Interpretive Description, this paper necessarily presents an interpretation of the experiences of people impacted by stroke. Both authors are allied health professionals (physiotherapist and speech‐ language therapist respectively), which may have focused the interpretation on rehabilitation‐related aspects of people's experiences. Moreover, both authors identify as Pākehā (New Zealand European) and thus bring this lens to their analysis. The key themes underpinning this paper were shared with participants, and feedback was discussed via a range of means over time, including through illustrated booklets, individual conversations and small group discussions. Further, data collection, initial familiarisation and early analytic discussions occurred in conjunction with a Māori stroke researcher. However, we acknowledge that this paper cannot represent the deeply nuanced understandings of well‐being across the diversity of both Māori and non‐Māori worldviews and experiences in Aotearoa.

## 3 Results

We spoke with 24 people with stroke and 13 family members from the North Island of New Zealand, predominantly from urban areas as classified using the Geographic Classification for Health (GCH), a system of defining people's health locations [21]. Twelve people with stroke and seven family members identified as Māori. Participants were aged between 34 and 80 years, with an average age of 54 years at the time of stroke. Most had experienced their stroke within the previous 5 years, resulting in a variety of self‐reported stroke impacts. The characteristics of participants with stroke are provided in Table 1, while characteristics of family members are provided in Table 2. Participants could self‐identify with more than one ethnicity, which is reflected in the tables below. Ethnicity data are presented using level one categories as per the Ethnicity New Zealand Standard Classification 2005, V2 [22].

**Table 1:** Characteristics of people with stroke.

Age at time of stroke	
– < 20	1 (1 Māori)
– 20–29	—
– 30–39	2 (1 Māori)
– 40–49	7 (4 Māori)
– 50–59	4 (3 Māori)
– 60–69	7 (3 Māori)
– 70–79	2
– > 80	1
Years post‐stroke	
– < 1 Year	10
– 1–4 years	5
– 5–9 years	5
– 10–14 years	1
– 15–19 years	—
– > 20 years	3
Self‐reported impacts of stroke	
– Physical impairments including hemiplegia, reduced balance and co‐ordination, sensory impacts	14
– Speech and language impairments	12
– Fatigue	8
– Cognitive impairments including impacts on concentration, memory and processing	5
– Stress and anxiety	2
– Agnosia	1
– Pain	1
Gender (self‐identified)	
– Male	11
– Female	13
Ethnicity (noting people self‐identified ethnicity and some selected > 1)	
– New Zealand European	11
– Māori	12
– Pacific peoples	1
– Asian	1
Residential location using GCH classification	
– Urban 1	17
– Urban 2	6
– Rural 1	1

**Table 2:** Characteristics of family members.

Gender (self‐identified)	
– Male	3
– Female	10
Ethnicity	
– New Zealand European	4
– Māori	7
– Asian	1
– Not given	1
Relationship	
– Spouse/partner	6
– Child	2
– Sibling	3
– Carer	1
– Parent	1

People with stroke *and* family were doing significant work to support their well‐being whilst receiving care from stroke services. We identified six interwoven forms of work which are presented under the categories of ‘inward work’ and ‘outward work’. Inward work involved sense‐making, emotional work and reconnecting work. Outward work involved navigating health services, advocating for needs, and doing rehabilitation. Relationships were a critical component of well‐being and included connections with whānau (wider kinship networks), family and friends; new relationships with health professionals; and connecting with others with stroke. Such relationships were central in supporting people to do both inward and outward work to support their own well‐being. Each participant's experience was unique, and the six areas of work were deeply interwoven. As such, it was not possible to identify a clear pattern of ‘work’ across recovery stages. However, while sense‐making, navigation, advocacy work and doing rehabilitation were evident across participants’ experiences, they were especially prominent during inpatient care and shortly after discharge. Emotional and (re)connecting work were evident throughout participant narratives, including, for some, many years post‐stroke.

**Figure 1:**
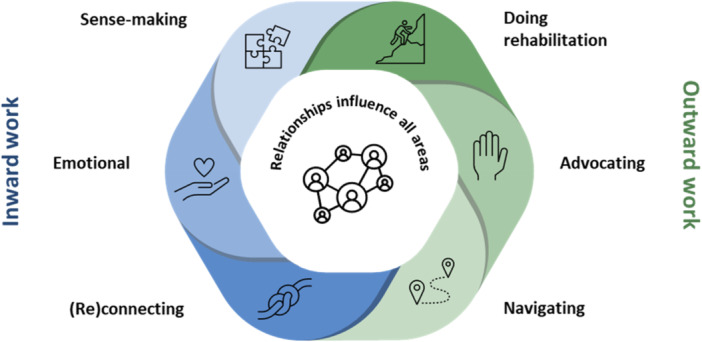
The six interwoven forms of work that people and family are engaged in to support well‐being after stroke.

Figure 1 provides a graphic representation of the results. While it specifies the six lines of work, it also shows how these are interconnected, and how relationships appeared to support and influence every area of work.

### 3.1 Inward Work

#### 3.1.1 ‘There Was So Much Uncertainty’—The Sense‐Making Work

People consistently spoke of the immense uncertainty of stroke. They described trying to make sense of what had happened, particularly in the early days of recovery. People wanted to understand the stroke and what might have led to it, in part to quell the fear of having another. They appreciated health professionals who explained information clearly and empathetically. This could support a sense of stability and reassurance.

There was this lady from the stroke team … She pretty much broke it down to me about what I had, showed me pictures, and explained to me how a stroke works … And it doesn't make any sense until someone explains it thoroughly and properly it's like, “wow ok”. That was really good, and really helpful. It was only at that time I thought “oh ok … I know what's happening”.

(Man with stroke, Pacific person)

However, many people described receiving generic, and at times conflicting information, which compounded feelings of distress and uncertainty. Some Māori participants found that information provided was not culturally appropriate, with one woman with stroke describing a lack of *‘understanding of whānau (wider kinship network) needs from a Māori perspective’*. This ultimately required them to seek out their own sources of information which reflected Māori worldviews, often through personal networks.

#### 3.1.2 ‘It Is a Gut Wrenchingly Emotional Time…We Should Be Allowed to Be Gut Wrenchingly Emotional’—The Emotional Work

People experienced a multitude of emotions after stroke. While not all were negative, a hallmark across experiences was a feeling of deep distress during time in care. People described undertaking significant emotional work to navigate this period. They spoke of trying to ‘sit with’ many changing, complex emotions. Many were navigating feelings of disconnection from themselves, from important others, and from things that had been meaningful to them.

It's like you're in another world of being protected, cared for, you don't understand it yourself…You want your life back again and you've got all this emotional stuff going on that you can't relate to because it's new, it's horrific, it's traumatic…And no‐one knows, you've completely changed from what you were prior to your stroke.

(Woman with stroke, Māori)

Several people described individual health professionals who recognised the emotional impacts of stroke and provided support. This was often through taking the time to get to know the person, and prioritising ‘sitting and talking’ when needed. However, most felt the depth of their distress was not seen by staff. Some alluded to feeling dismissed when told by staff that their emotions were ‘normal’. Moreover, the busyness of staff and a focus on physical recovery meant that people often did not feel comfortable sharing their emotions. This could compound the emotional burden people carried and contributed to people feeling alone and unheard.

Sometimes we tend to not speak the truth because we feel like lots of expectancy, sometimes we are afraid, or I felt like I was afraid. Because they [community rehabilitation team] would be “oh you are looking good” after maybe the second, third week they would say “oh you are looking much better”. I felt physically better, but it doesn't mean to say that I was mentally better. I just sometimes went “oh I am good, I am ok”.

(Man with stroke, Pacific person)

There was seen to be a widespread lack of counselling and emotional support available. Two Māori participants spoke explicitly of the need for counselling grounded in a Māori worldview that includes whanaungatanga (building connections), wairua (spiritual sense) and hinengaro (mental well‐being). They, along with many other Māori participants, discussed the lack of recognition of their worldview as especially detrimental to well‐being.

#### 3.1.3 ‘Have I Still Got Value, Can I Still Do Things, Have I Still Got a Life?’—The (Re)Connecting Work

People were reflecting on how the stroke might impact their identity, their relationships and what they found important in life. They tried to develop a coherent narrative of their past, present and possible future.

‘When I had the stroke then I had to bloody retest and try and figure out who I am and what I am. You know, what kind of knowledge and skills have I got?’

(Man with stroke, Māori)

(Re)connecting with areas of meaning seemed to sustain people through this process. For example, everyone spoke of relationships and meaningful roles within whānau (wider kinship network) or community that provided an anchor point through recovery. Many Māori participants expressed a sense of grounding in their cultural identity, and the importance of wairua (spiritual sense), reo (Māori language), te taiao (the natural environment), or connecting with places of meaning. However, (re)connecting with such areas could take significant effort, as one woman with stroke described finding herself *‘having to relearn everything’*, and others spoke of fighting feelings of embarrassment and shame. Through their knowledge of the person with stroke, family and friends often fostered connection to what was meaningful. Māori family members in particular supported well‐being and upheld mana (inherent standing) in ways that services did not; by respecting tikanga (cultural practices), limiting visitors to protect one's mana, or providing holistic support from a Māori worldview.

‘That's what's held her together. Sometimes it's just been by the skin of her teeth, but it's been something, you know, that counselling [I gave] to her wairua (spiritual well‐being) side, to her hinengaro (mental well‐being) side…’

(Husband of woman with stroke, Māori)

Staff in stroke services were not seen to recognise the importance of this reconnecting work, and the value of attending to areas of meaning. In fact, services could act to further disconnect people from the things that were important.

‘[Dad] turned a lot to his taonga puoro (traditional musical instruments) …You know, no health professional would've ever said to him, “Now how can we support you to be able to make your taonga puoro again?”…Things that were important to him were not factored into any of their rehabilitation sort of programmes’.

(Daughter of man with stroke, Māori)

### 3.2 Outward Work

While making sense of the stroke, grappling with a multitude of emotions and trying to reconnect with areas of meaning, people were also navigating complex health systems and engaging in the day‐to‐day activities of rehabilitation. Having a clear understanding of healthcare processes, feeling listened to, and having autonomy over healthcare choices helped people to maintain a sense of control over their recovery journey. Moreover, seeing progress in rehabilitation *and* how this connected to wider areas of meaning in life could support people to look to the future with hope.

#### 3.2.1 ‘We Don't Get Told in Hospital What's Available to Us While We're There Let Alone When We Leave!’—The Navigating Work

People felt a greater sense of control over their care when they understood the healthcare environment and care processes. They appreciated knowing what would happen, when and why. They wanted information on what support was available and how to access it at different stages of care. However, hospital and community services were often confusing, which increased the work people needed to do to make sense of health. The rationale for assessments, investigations and interventions was not always shared, requiring people to deduce what was happening and why. Medical terminology could marginalise people and family members. As one woman with stroke explained, this left people feeling they were not ‘*included in the conversation’* and reduced their autonomy. Family often became ‘navigators’, with many dedicating considerable time to ‘chasing’ staff or seeking out service support. Having an understanding of the health system appeared to facilitate this work. People frequently spoke of the need for a support person to ease the additional burden of navigating service processes.

‘It's like you need, oh like a ward nana or somebody to sit and just go through it all with you…To sit down with a partner or the other person and just check everything's alright and say, “well this is available and this is available” to try and help you through the situation…It's just little things that make your life, well, our lives easier because it's such a traumatic time.’

(Man with stroke, New Zealand European)

#### 3.2.2 ‘[My Aunty] She Pushed Buttons and Stuff, and She Made Sure That People Heard What I Wanted’—The Advocating Work

Woven throughout participant narratives was the importance of being heard and listened to by health professionals. When care felt like a process of shared problem‐solving about what would work best for the person and their family, this upheld mana (inherent standing) and fostered choice and control.

‘The nurse would say “okay so if we got you the wheelchair or if we showered you earlier in the morning, we've got this staff member that starts at this time – would that make you happier?” And my husband would go, “actually yes, that would make a huge difference”. So, she was very good at recognising the simple things that make a world of difference.’

(Wife of man with stroke, New Zealand European)

However, a significant number of participants felt their perspectives were not sought or were overridden by health professionals.

‘They have their best intentions but it's like the old “well I know what you need, and I'll give it to you ‘cause I know what you need”. No‐one asked me “what do you need or what would you like?” ‘Cause I wanted to go swimming, and they said, “oh no you can't do that”. And I went “oh, ok”. “You can just go and see the psychologist’’’.

(Woman with stroke, Māori)

Many family members found themselves advocating strongly for their loved one's needs. This experience was compounded for many Māori who did not feel that their needs were met within Western‐oriented health services. Several put strategies in place to protect their loved one within this space. Indeed, some felt that the hospital was not a healing environment, and thus, once medically safe to do so, arranged for their loved one to move home where they felt better placed to meet their needs.

At an individual level, several people with stroke described wanting opportunities to build self‐reliance, particularly during inpatient care. However, they found that service processes stifled their autonomy over their recovery. Some found ways to overcome this, but this was not always welcomed by staff.

‘A major issue was having someone there. You had to depend on them, whenever they were ready to take you to do certain things…Sometimes I'd actually break the rules, because I'd have to wait for a nurse and then I'd be sitting on the bed for an hour, maybe two hours till I could get somebody…So that was probably, I got out of hospital earlier than what I should, because of what I did’.

(Man with stroke, Māori)

#### 3.2.3 ‘It Was Sheer Hard Work’—The Work of Doing Rehabilitation

Throughout care, people were engaged in the work of doing rehabilitation. This was orientated toward managing the body in the here and now. People were practicing functional tasks, engaging in therapy activities and working towards short‐term goals. This had positive impacts on well‐being by providing structure and fostering a sense of progress.

‘That's what I lived for was the therapy sessions because if you have a bad day and then you have a good session, it brings you up. You've got that sense of achievement that you're doing something for your recovery’.

(Man with stroke, New Zealand European)

Several participants described feeling buoyed by the encouragement of staff in this process, with one man saying, ‘*Her [physiotherapist] strength became my strength’ (New Zealand European)*.

However, rehabilitation was commonly short‐term focused, restricted by the ‘rules of the ward’ and did not necessarily support people to see how rehabilitation activities could connect to the wider things that mattered for well‐being. People described a sense of being moved through services as though on a conveyor belt. One Māori woman with stroke described feeling ‘*all broken up’* by Western notions of health and well‐being. This contradicts Māori models of hauora (health) which are holistic and multi‐dimensional. Indeed, health environments, processes and practices that did not recognise nor reflect Māori ways of knowing, being and doing compounded the work Māori participants needed to do for their recovery.

Discharge from care could be challenging when the work of doing rehabilitation was heavily focused on the short‐term and achieving basic function, without connecting to wider supports for well‐being. Some people felt cast adrift or, as one man with stroke described, feeling ‘*dumped’* out of hospital as service input came to an end, and daily structures and overt progress markers were removed. People were thrust into a new stage of recovery where they had to figure out what the ‘work of rehabilitation’ might now look like. This, in turn, could exacerbate the emotional work of people and family as many grappled with feelings of isolation and uncertainty. Similarly, the burden of navigating community services and advocating for one's needs could increase as people tried to seek out the support they needed.

‘It was just like ok you've done your six weeks or eight weeks or six months or however long it was and tick and off you go and that's what I said to them, “Oh I ticked that box and now I'm out of here, what do I do now? What do I do?” And I didn't know what to do’.

(Woman with stroke, Māori)

#### 3.2.4 The Critical Component: *‘*Everything Is About Relationships’

Relationships were central in supporting people to do the inward and outward work to support their own well‐being. Such relationships incorporated both new and old— connections with whānau, family and friends; new relationships with health professionals; and connecting with others with stroke.

Family, whānau (wider kinship networks) and friends often held deep knowledge of the person with stroke. They supported sense‐making and helped the person with stroke to reconnect with areas of meaning in their lives. One man with stroke described how his wife could see *‘the real me’* and provided emotional support and grounding. Family members frequently took up the roles of ‘navigator’ and ‘advocate’, seeking out supports and advocating for the wishes of their loved one. This could reduce the burden on the person with stroke during their time in care, as one man with stroke described, *‘If I needed help, well my kids were there to help me’.*

Strong relationships with health professionals often became a source of stability, guidance and encouragement as people undertook the work of rehabilitation, and the work of well‐being more broadly. Such relationships were also valued by family members – with the wife of a man with stroke describing the comfort of knowing their healthcare team *‘really cared’*. Moreover, of great significance for some people were the new friendships they developed with others with stroke. These relationships fostered *collective* sense‐making, which helped people to come to terms with what had happened, find a sense of belonging, or strength and purpose to move forward. Such connections were of particular importance for many Māori participants, who described a sense of connection not felt in other spaces:

‘I just felt more comfortable to know that there were other Māori out there going through the same stuff as me in every way you know, in support ways, hospital ways, family ways, they were all going through the same stuff. That was really good to know’.

(Woman with stroke, Māori)

Not only did people with stroke find support within such relationships, they too *provided* support. These relationships were reciprocal. Participants described protecting and caring for their loved ones, sharing motivation and encouragement with others in hospital, or using their experience to help others with stroke. However, relationships were also complex. Many described feeling supported while simultaneously managing altered relational dynamics. One woman with stroke spoke of the challenge of connecting with others while feeling *‘completely changed’*. Others alluded to similar feelings, particularly in the context of communication impairment or for those with a ‘mild’ stroke. Family members spoke of supporting their loved one while managing their own grief and taking on new roles within the family. Relationships were fundamental, serving as both a vital support through the ‘work of well‐being’ *and* as a deeply meaningful area of life that required their own ‘relational work’.

## 4 Discussion

This paper makes visible the multiple layers of work that people were actively doing to support their well‐being after stroke. Literature often suggests that people have low levels of activity in rehabilitation services, commonly attending to physical and/or communication activity levels [23, 24, 25]. However, our results surface a different form of activity that people are engaged in ‐ the activity required to manage their well‐being. This is often done alone and in ways that can be invisible unless staff explicitly engage with people about the well‐being work they are doing. Like Costa and colleagues [26], who have problematised the notion of inactivity, our work shows that people are active in ways that may not be recognised nor supported in the healthcare context. As such, health professionals and researchers need to have broader conceptualisations of what work people do—and indeed, what work is seen and recognised as legitimate. In fact, healthcare environments, structures and processes can drive particular types of (in)action and can increase the burden of unseen work that people must do to sustain their well‐being. Several authors have described, for instance, hospital policies and environments that inadvertently discourage activity and limit autonomy [23, 27] or constrain people's ability to engage in meaningful activities [26]. Our participants alluded to how service structures and processes could exacerbate the burden of work they carried, for instance through inconsistent information provision and confusing service processes, by not welcoming nor supporting a person's relational networks, and through a lack of culturally safe care. This aligns with the work of Sheehy et al. [28] who make clear the additional burden often carried by Māori when engaging with physical rehabilitation services. The authors describe the work required of Māori to navigate the many ‘cultural collisions’ (p3) between Te Ao Pākehā (the New Zealand European world) and Te Ao Māori (the Māori world). Such work includes taking up advocating, navigating and cultural and spiritual support roles for whānau (wider kinship group) members where services do not provide appropriate support. This additional burden has implications both for collective whānau healing, and for the individual accessing rehabilitation.

Some readers may query whether the work of well‐being simply constitutes self‐management after stroke. Traditional self‐management approaches focus on managing the impacts of the stroke itself and often relate to functional improvement and physical independence [29]. Our findings suggest that well‐being work is broad and relational, more akin to contemporary discussions of self‐management [30, 31]. These position self‐management as inherently grounded in relationships where people are supported to reflect on and make sense of what has happened, and to envisage possible futures [30, 31]. Moreover, the work of well‐being is sometimes compounded by healthcare processes and the ways in which health professionals work, drawing parallels with the concept of treatment burden [12]. In this way, it may be argued that well‐being work sits at the intersection of treatment burden and contemporary notions of self‐management. This presents a tension—well‐being work is inherently relational, but its need can be exacerbated because of the experiences people have in services. To address such a tension requires health professionals to be able to take time for supportive conversations, to recognise the work that people are doing, and to be able to provide them with support as they navigate life after stroke [31, 32]. Further, it is vital to not only recognise the work that people are already doing but also to recognise the cognitive and emotional landscape that they are navigating. Incorporating such a focus into stroke care requires a fundamental shift in the dominant rehabilitation model, which focuses on physical recovery and the health professional as expert [33], towards genuine person‐centred care [34].

The experiences of our participants provide a unique insight into both the resources people draw on and the challenges they face when navigating well‐being after stroke. Our previous work has shown the challenges health professionals perceive in addressing well‐being [9]. This paper, centring the perspectives of those living with stroke, highlights areas of opportunity where health professionals and stroke services may reduce the burden of work people carry and support them to navigate life after stroke. While some of these areas require structural supports, many are interactionally achieved and reflect ways of working that can be built into routine service provision. For example, participants made clear the importance of friends, family and whānau (wider kinship networks) in sustaining their well‐being. Similarly, every participant described drawing on areas of personal meaning to support their post‐stroke journey. Health professionals have the potential to either recognise and foster such resources, or to disregard or diminish them. By fostering resources that people already hold, such as actively supporting people to engage in activities, roles, relationships, cultural practices and social contexts that support their well‐being from early in care, health professionals may help to ease the work people undertake to support their well‐being. Our findings support the argument for flipping the discourse of stroke care from a short‐term, deficit focus, to one of strengths, reconstruction and growth [11, 15, 35]. This concept resonates with messages from Māori in our study who highlighted that stroke services are but a small part of post‐stroke recovery. Growth happens outside of services and over time, but services can help to recognise, foster and provide the conditions for growth. By acknowledging and supporting the depth of work that people do and fostering the strengths and resources they draw on for their well‐being, health professionals and service leaders may set a foundation to better support people's well‐being both in the moment and long‐term—beyond the timeframe of stroke services.

Grounded in the perspectives of people and family impacted by stroke, this paper offers insights into the complex ways in which people work to sustain their well‐being after stroke, and the influence of services in this process. Our use of NPT enabled us to attune to the work people were doing to support their well‐being. While more commonly used in the implementation and evaluation of complex interventions [18, 36], NPT proved a useful device to make visible the often‐unseen factors that impact a person's experience of care. Stroke care and rehabilitation are commonly task‐focused and orientated toward discharge. As such, there are many small moments of care that go unrecognised. These elements of care are essential but are not standardised nor set out in protocols, often rendering them invisible. NPT provided a useful way of surfacing such elements.

The study has several limitations which must be acknowledged. Most participants were under 65 years of age, and all from the North Island, which is not reflective of the stroke population in Aotearoa. Many participants had experienced significant disruption to their working lives, and several had young families at the time of stroke. As such, the relatively young age of participants may have influenced the types of work described in our analysis. In addition, while this research included the voices of Māori and New Zealand European people impacted by stroke, we had few Pacific peoples and people of Asian ethnicity within the study. These groups are significantly impacted by stroke [1]; thus, our findings do not reflect the many unique and differing experiences of stroke survivors across the country. It would be beneficial to explore these valuable perspectives in future research.

## 5 Conclusion

This research provides insight into the multiple ways in which people work to support their well‐being after stroke. The findings shed light on how stroke services can either add to or reduce the burden of ‘well‐being work’ that people carry, therefore highlighting areas of opportunity where health professionals and stroke services may focus efforts to better support well‐being. Health professionals can acknowledge the work that people are doing and foster the resources they already hold, such as actively supporting people to engage in activities, roles, relationships, cultural practices and social contexts that support their well‐being from early in care. In doing so, health professionals and others in the stroke care system may support a strong foundation for people's long‐term well‐being after stroke.

## Author Contributions

**Claire Ibell‐Roberts:** data curation, formal analysis, project administration, writing – original draft, writing – review and editing, investigation, methodology, conceptualisation. **Felicity Bright:** conceptualisation, methodology, investigation, formal analysis, supervision, funding acquisition, project administration, writing – review and editing.

## Ethics Statement

This research was approved by the Auckland University of Technology Ethics Committee (21/223).

## Consent

The authors have nothing to report.

## Conflicts of Interest

The authors declare no conflicts of interest.

## Data Availability

Ethical approval does not allow the authors to share the data.

## Appendix A Indicative questions for semi‐structured interviews

**Māori stream**

1. Well‐being after stroke
  a. Can you tell us how things have been for you since you had your stroke?
  b. People often talk about the importance of well‐being. What does well‐being mean to you?
  c. Can you share your experience of well‐being after stroke?
  d. People often talk about the importance of *psychosocial* well‐being after stroke. What do you think of when you hear that term? Are there other kupu (words) that you think are more appropriate?
  e. Do you feel you are experiencing psychosocial well‐being at the moment? Why or why not?
  f. When you think about yourself just after you had the stroke, what was most important in helping your well‐being at that time?
  g. How does wairua connect with well‐being?
  h. What role do whānau play in your well‐being?

**Stroke services**

a. Can you tell me about the care you had after your stroke? (Map out care on a piece of paper—services, clinicians, gaps in care. Probe for care practices and processes such as family meetings, new therapists, referrals to other services

**Psychosocial well‐being after stroke**

a. When you think about how the stroke services supported your well‐being, what went right, and what went wrong?
b. When we look at each step in your care, can you tell me how the staff supported you with each of these areas?
c. Was there any particular person who you felt helped? If so, what did they do?
d. Were there times where services or staff focused on other things instead? Were there times when they could have looked more at supporting you to live well after stroke? Are there some examples you could share?
e. Could you tell me about all the things you and your whānau have done to support your well‐being?

**Doing things differently**

a. Other than stroke services, what helps or could help your well‐being?
b. If you felt your well‐being was low, where would you go for help?
c. In an ideal world, how could services better support your psychosocial well‐being?
d. Is there anything services and clinicians could do differently to support other people who experience strokes in the future?

**Non‐Māori stream**

1. Well‐being after stroke
  a. Can you tell us how things have been for you since you had your stroke?
  b. People often talk about the importance of well‐being after stroke. What do you think of when you hear that term?
  c. People often talk about the importance of *psychosocial* well‐being after stroke. What do you think of when you hear that term?
  d. Do you feel you are experiencing psychosocial well‐being at the moment? Why or why not?
  e. When you think about yourself just after you had the stroke, what was most important in helping your well‐being at that time?
2. Stroke services

a. Can you tell me about the care you had after your stroke? (Map out care on a piece of paper—services, clinicians, gaps in care. Probe for care practices and processes such as family meetings, new therapists, referrals to other services

3. Psychosocial well‐being after stroke

a. When you think about how the stroke services supported your psychosocial well‐being, what went right, and what went wrong?
b. When we look at each step in your care, can you tell me how the staff supported you with each of these areas?
c. Was there any particular person who you felt helped? If so, what did they do?
d. Were there times where services or staff focused on other things instead? Were there times when they could have looked more at supporting you to live well after stroke?
e. Could you tell me about all the things you and your family have done to support your well‐being?

4. Doing things differently

a. Other than stroke services, what helps or could help your psychosocial well‐being?
b. If you felt your well‐being was low, where would you go for help?
c. In an ideal world, how could services better support your psychosocial well‐being?
d. Is there anything services and clinicians could do differently to support other people who experience strokes in the future.

## Appendix B Framework for analysing the work of people and family in managing well‐being after stroke

**Table:**

Sense‐making work	Relational work	Enacting work	Reflection work
*Making sense of stroke, how it can be managed, and what the future might look like*	*Interpersonal aspects of work to manage well‐being*	*Enacting management strategies/organising and enacting collective tasks (as per Makela)*	*Reflecting on change and implications for managing well‐being into the future*
Understanding what has happened and why, and the impacts of the stroke; differentiating between tests, treatments and roles of health professionals	Determining and (re)establishing support networks through engaging with whānau (wider kinship networks), family, friends, community, health professionals	Coping with impacts of stroke while managing day‐to‐day life	Adapting to change in stroke impacts, well‐being, broader life circumstances as they occur
Help from others in understanding what is happening	Making arrangements for help and negotiating supports with others	Integrating stroke into social circumstances, determining and drawing on resources to meet well‐being needs	Drawing on others to support reflection on, and adaptation of management of well‐being over time
Achieving a personal understanding of the stroke and its management in ways that are personally meaningful	Personal planning of one's own management of stroke and well‐being	Undertaking treatment and rehabilitation, actions taken to engage in meaningful roles and integrate stroke into life	Assessing individually whether changes to manage well‐being are required, identifying priorities
Understanding implications of stroke in personal context and determining when and how to seek help	Seeking legitimation and reassurance from others regarding concerns or ways of managing	Using facilitators and dealing with barriers to managing well‐being	Developing ways to continue to adapt to changes to impacts of stroke, well‐being, broader life circumstances into the future
